# Management and characteristics of patients suffering from *Clostridiodes difficile* infection in primary care

**DOI:** 10.1080/13814788.2021.1998447

**Published:** 2021-11-10

**Authors:** Maria Klezovich-Bénard, Frédérique Bouchand, Elisabeth Rouveix, Pierre L. Goossens, Benjamin Davido

**Affiliations:** a Département de Médecine Générale, l’Université de Versailles Saint Quentin en Yvelines, France; b Pharmacie Hospitalière, Centre Hospitalier Universitaire Raymond Poincaré, AP-HP, Garches, France; c Service des Maladies Infectieuses, Centre Hospitalier Universitaire Raymond Poincaré, AP-HP, Garches, France; d Yersinia Research Unit, Institut Pasteur, Paris, France

**Keywords:** *C. difficile*, primary care, general practitioner, infection, management

## Abstract

**Background:**

*Clostridioides difficile* infection (CDI) is rising and increases patient healthcare costs due to extended hospitalisation, tests and medications. Management of CDI in French primary care is poorly reported.

**Objectives:**

To characterise patients suffering from CDI, managed in primary care and describe their clinical outcomes.

**Methods:**

Retrospective observational study based on survey data among 500 randomly selected General Practitioners (GPs) surveyed in France from September 2018 to April 2019. GPs were asked to complete a multiple-choice questionnaire for each reported patient presenting a CDI. Responses were analysed according to clinical characteristics. Treatment strategies were compared according to the outcome: recovery or recurrent infection.

**Results:**

Participation rate was 8.6% (*n* = 43/500) with two incomplete questionnaires. Data from 41 patients with an actual diagnosis of CDI were analysed. Recovery was observed in 61% of patients with a confirmed diagnosis of CDI. In the recovery group, this was exclusively a primary episode, most patients (72%) had no comorbidities, were significantly younger (*p* = 0.02) than the ones who relapsed and 92% were successfully treated with oral metronidazole. Duration of diarrhoea after antimicrobial treatment initiation was significantly shorter in the recovery group (≤ 48 h) (*p* = 0.03). Cooperation with hospital specialists was reported in 28% of the recovery group versus 87.5% of the recurrent group (*p* = 0.0003). Overall, GPs managed successfully 82.9% of cases without need of hospital admission.

**Conclusion:**

GPs provide relevant ambulatory care for mild primary episodes of CDI using oral metronidazole. Persistent diarrhoea despite an appropriate anti-Clostridiodes regimen should be interpreted as an early predictor of relapse.

KEY MESSAGES

- Oral metronidazole appears acceptable for the treatment of a first episode of *C. difficile*
- Patients with persistent diarrhoea after 48 hours of appropriate anti-Clostridiodes therapy should be addressed to specialist
- Patients should be asked if they are using over-the-counter laxatives or other medications

## Introduction

*Clostridioides difficile* infections (CDIs) are commonly associated with healthcare facilities and mostly known as antibiotic-associated diarrhoea affecting particular patients with risk factors [1,2]. CDI has also been increasingly reported outside of hospital, including in community and nursing homes settings [3], where infection may be diagnosed and treated without hospitalisation. Due to the emergence of CDIs, general practitioners (GPs) are urged to manage this infection in their daily practice [4]. Indeed, *C. difficile* also affects patients considered at low risk for developing a CDI, such as young patients without comorbidities and those without previous hospitalisation or prior exposure to antimicrobials agents [5]. For instance, in France, the actual burden of CDI at the primary care level is still underestimated because of a lack of clinical suspicion or a lack of sensitivity of methods used for toxin detection [6].

The clinical variations of CDI range from asymptomatic carriage through diarrhoea, from pseudomembranous colitis to toxic megacolon [7]. Several laboratory tests are currently available to diagnose CDI but only the detection of toxin is critical for clinical diagnosis of CDI [8].

The primary treatment has been antibiotics such as metronidazole and vancomycin. Fidaxomicin was approved by the European Medicine Agency in 2011 and is a better treatment option than vancomycin for recurrent CDI [9] throughout Europe. Either vancomycin or fidaxomicin is now recommended over metronidazole for an initial episode of CDI. However, providing those drugs to GPs is not possible, leading to use of metronidazole as the first line therapy available in ambulatory care for an initial episode of non-severe CDI [10].

Due to limited hospital resources, primary care plays a crucial role in reducing the inevitable flow of numerous patients visiting emergency departments [11]. Yet, although the overall impact of primary care on clinical outcomes in patients with community CDI has already been investigated in other European countries [12–14], few studies have reported in France. Recent studies concerning the United States and Europe suggest that community-acquired infections account for about 27–41% of all cases of CDI [15,16]. In Europe, in a multicentre point-prevalence study, toxigenic *C. difficile* has been identified as the third most common bacterial cause of community-associated diarrhoea (2.4%), after *Campylobacter spp* and *Salmonella spp* [6].

This study aims to collect estimates on the situation of CDI at primary care level in France by characterising features of outpatients suffering from CDI, treatment strategies used and describing their clinical outcomes and establishing whether the management of CDIs is appropriate or requires the help of hospital specialists.

## Methods

### Study design and setting

We conducted a retrospective observational study. Clinical and demographic characteristics were obtained through an anonymised online questionnaire survey of GPs in France from September 2018 to April 2019 concerning their management of CDI. A database of community-based GPs containing valid email and postal addresses of physicians who consented to receive marketing offers were obtained from a private marketing company (Ideactif) registered under the National Commission on Informatics and Liberty (CNIL) number 1666522-v0. We invited 500 random GPs practising in the community in France. We excluded those practicing exclusively complementary and alternative medicine to ensure of being the most representative of the mainstream healthcare.

### Clinical data collection

Considering CDI is a rare disease in ambulatory care, each GP was asked to complete an online questionnaire (Supplementary data) concerning the last patient seen in consultation in the previous 24 months to obtain the most total number of possible responses. Data extracted from electronic medical charts were age, sex, diarrhoea duration, clinical appearance of CDI, presence of severity factors, antecedent of recent hospitalisation, previous use of antibiotics, NSAIDs, laxatives, PPIs, method of laboratory detection, specific therapy prescribed, cooperation with hospital specialists and clinical outcome.

To compare characteristics of individuals, patients were divided into two groups according to clinical issue status: recovery or recurrent infection.

### Definitions

- Confirmed *C. difficile* infection was defined by the clinical presence of diarrhoea and a positive finding of *C. difficile* toxins in stool samples (by PCR or immune assay) [17].
- Long-lasting diarrhoea was defined by the persistence of symptoms (≥ 3 unformed stools in 24 hours) more than three days under symptomatic treatment using anti-diarrheal drugs regardless of the outcome.
- Recovery was defined by resolution of symptoms at the end of specific treatment.
- Recurrent *C. difficile* infection was defined by reappearance of symptoms within four weeks after treatment completion [18,19].

### Statistical analysis

Non-parametric tests were used to compare quantitative variables. Fisher's exact test was used when appropriate to compare proportions (*N* < 30) or Chi-square test for categorised variables using GraphPad Prism v.7.0 (GraphPad Software Inc., La Jolla, CA). Statistical significance was defined as *p* < 0.05.

## Results

### Patient characteristics, symptoms and risk factors

Participation rate was 8.6% (*n* = 43/500) with two incomplete questionnaires. Data from 41 patients with a confirmed diagnosis of CDI were used in the analyses. The large majority of CDIs (90%) were diagnosed by searching for *Clostridium difficile toxin* B or A by enzyme*-*linked immunosorbent assays (ELISA). A subsequent PCR was performed once for a patient who required hospitalisation.

Initial clinical characteristics of the patients at the first presentation to the GP are presented in Table 1 according to clinical issue status (i.e. recovery or recurrent infection).

**Table 1.** Characteristics of patients with confirmed *Clostridiodes difficile* infection according to clinical issue status.

	Recovery	Recurrent infection	*p-*value
Total – no.(%)	25 (61)	16 (39)	–
Age – no.(%)**:			
<30 yr	2 (8)	1 (6.25)	
30–65 yr	19 (76)	6 (37.5)	**0.02**
>65 yr	4 (16)	9 (56.2)	
Female sex – no. (%)*	15 (60)	10 (62.5)	0.99
Primary infection – no. (%)*	25 (100)	12 (75)	**0.02**
Symptoms of current *C. difficile* infection – no. (%):			
Febrile diarrhoea and/or inflammation*	9 (36)	6 (37.5)	0.99
Haemorrhagic and/or diarrhoea with mucus*	12 (48)	5 (31.2)	0.34
Dyspepsia and/or abdominal pain*	8 (32)	7 (43.8)	0.52
- Long-lasting diarrhoea despite anti-diarrheal drugs**	13 (52)	10 (62.5)	0.51
Past medical history – no. (%):			
Immunodepression*	1 (4)	2 (12.5)	0.55
Inflammatory bowel disease*	1 (4)	3 (18.8)	0.28
Previous hospitalisation*	5 (20)	8 (50)	0.1
No past medical history*	18 (72)	3 (18.8)	**0.001**
Recent history of antibiotic use – no. (%):**	17 (68)	9 (56.2)	0.44
Amoxicillin	5 (20)	3 (18.8)	0.58
Amoxicillin/clavulanic acid	7 (28)	3 (18.8)	
Third-Generation Cephalosporins	1 (4)	2 (12.5)	
Other	4 (15)	1 (6.25)	
Use of proton pump inhibitors – no. (%)*	7 (28)	4 (25)	0.99
Use of non-steroidal anti-inflammatory drugs – no. (%) *	6 (24)	0 (0)	0.06
Use of laxatives/ antidiarrheal agents – no. (%)*	1 (4)	5 (31)	**0.03**

*Univariate analyses according to Fisher’s exact test or Chi-square test** for categorised variables.

Overall, half of the patients were without past medical history (*n* = 21; 51%). Long-lasting diarrhoea was the most frequent clinical presentation (*n* = 23; 56%). Recent or current intake of antibiotics was observed in 26 patients (63%).

Patients in the recovery group, compared to the recurrent group, presented solely a primary episode of CDI (100% vs. 75%; *p* = 0.02), were significantly younger (*p* = 0.02) with no comorbidities (72% vs. 19%; *p* = 0.001). The use of laxatives and/or antidiarrheal agents was more frequently reported in the recurrent group (31% vs. 4%; *p* = 0.03).

### Treatment strategies, diarrhoea duration and outcomes

Results are summarised in Table 2. When CDI occurred under antibiotic regimen, discontinuation of antimicrobial therapy was conducted only in 23% of cases (*n* = 6).

**Table 2.** Strategies, therapeutic methods used and duration of diarrhoea in the two groups after the initiation of specific treatment.

	Recovery	Recurrent infection	*p* value
Total – no.(%) 25 (61) 16 (39) -			
Specific drug prescription – no. (%):**			
Metronidazole	23 (92)	11 (68.8)	**0.001**
Vancomycin	0 (0)	7 (43.8)	
Fidaxomicin	0 (0)	2 (12.5)	
Diarrhoea duration after treatment initiation – no. (%):**			
24 h	1 (4)	0 (0)	**0.03**
48 h	14 (56)	2 (12.5)	
> 48 h	8 (32)	12 (75)	
Missing data	2 (8)	2 (12.5)	
Hospital admission – no. (%)*	1 (4)	6 (37.5)	**0.01**
Probiotics prescription – no. (%)**	3 (12)	1 (6.2)	0.54
Discontinuation of causal antibiotic – no. (%)**	4 (16)	2 (12.5)	0.75
Severity risk factors – no. (%)*	9 (36)	11 (68.8)	**0.02**
Cooperation with the hospital specialists – no (%)*	7 (28)	14 (87.5)	**0.0003**

*Univariate analyses according to Fisher’s exact test or Chi-square test** for categorised variables.

In the recovery group, oral metronidazole was successfully used as single therapy, compared to the recurrent infection group (*p* = 0.001) where other regimens, especially vancomycin, were necessary (44%).

The reported duration of diarrhoea after starting an appropriate treatment by metronidazole was significantly shorter (< 48 h) in the recovery group than in the recurrent group (*p* = 0.03).

A significantly higher cooperation rate with a hospital specialist was observed in the recurrent group (87.5% vs. 28%; *p* = 0.0003). Overall, seven patients (17.1%) were admitted to the hospital, among six patients (37.5%) in the recurrent group to discuss a new therapeutic option in contrast with only one patient (4%) in the recovery group but for intravenous rehydration (*p* = 0.01).

## Discussion

### Main findings

Recovery was observed in 61% of patients with a confirmed diagnosis of CDI. In the recovery group, most patients (72%) had no comorbidities, were significantly younger (*p* = 0.02) than those who relapsed and 92% were successfully treated with oral metronidazole. Also, laxatives and/or antidiarrheal agents were commonly associated with relapse (*p* = 0.03). Duration of diarrhoea after antimicrobial treatment initiation for *C. difficile* was significantly shorter in the recovery group (≤ 48 h) (*p* = 0.03).

### Interpretation

We have noticed that GPs mainly take care of young patients presenting primary mild underlying CDI. Indeed, successful recovery by a single oral metronidazole therapy was more frequently reported for the first episode of CDI, especially among young patients with no past medical history and mild underlying conditions. According to data issued by Dutch laboratories, CDI is a relatively frequent disease in general practice [20].

In fact, to a large extent, other studies have already shown that patients aged over 65 years often have multiple illnesses and are more likely to be directly hospitalised without interacting with primary care physicians, even for common diagnoses not generally considered requiring specialist’ care [21].

Yet, a treatment based on vancomycin or fidaxomicin administration was often needed for patients with severe underlying conditions. Therefore, teamwork between the generalist and specialist was necessary for such complex situations and typically for the management of recurrent CDI. This is concordant with a previous study reporting that 30% of patients consulted their GPs before visiting the emergency department [22], illustrating the need for collaboration between ambulatory and hospital care.

Until 2014, in mild-to-moderate cases, oral metronidazole was adequate first-line therapy and vancomycin was advised for patients with severe CDI [23]. However, two clinical trials published in the same year found metronidazole inferior to vancomycin [24].

We observed that the use of laxatives and/or antidiarrheal agents was commonly associated with recurrent CDIs. A previous study suggested that a prolonged time to resolution and recurrences were associated with the concomitant use of laxatives and/or antidiarrheal agents with antimicrobials [25]. The pathogenesis of recurrent CDI is still poorly understood. Various studies have underlined that increased abundance of *Enterobacteriaceae* and *Streptococcaceae* species could play a role in the likelihood of developing a CDI [26].

### Strengths and limitations

The main limitation is the observational retrospective nature of the study, with a small sample size as testing for *C. difficile* remaining modest in ambulatory care. Some other factors are associated with difficulty in recalling past events that may have also contributed to memorisation biases. Also, some could argue that we did not perform multivariate analyses due to the limited sample size.

Although it can be disputed that the invited GPs in France were not representative of the whole organisation and offer of care in European countries, we experienced only a few uncompleted questionnaires.

### Implications for research and practice

Based on our observations, we believe that a longer duration of diarrhoea under an appropriate anti-infective therapy against CDI (> 48 h) could be considered as a predictive indicator of a possible relapse. To our knowledge, this criterion has not yet been described in the literature. It could help to target patients at risk of failure and encourage GPs to contact hospital specialists rapidly.

Although vancomycin or fidaxomicin are recommended over metronidazole for an initial episode of CDI [10], metronidazole is still the less expensive treatment of choice and is available outside the hospital in pharmacy and also contributes to limit the development of vancomycin-resistant organisms [24].

It is important to note that the only effective strategy for preventing recurrent CDI remains reasonable use of drugs, including antibiotics and other medications taken over-the-counter. Moreover, it should be kept in mind that prevention of contamination by healthcare workers is a hot topic, especially for *C. difficile*, which requires handwashing with soap whereas everybody has begun to use hydroalcoholic gels since the COVID-19 pandemic.

## Conclusion

Our study shows that GPs provide relevant ambulatory care for mild primary episodes of CDI using oral metronidazole. Persistent diarrhoea despite an appropriate anti-Clostridiodes regimen should be interpreted as an early predictor of relapse.

Management by GPs in collaboration with hospital specialists helped the majority of patients to avoid unnecessary and costly hospital admissions. Therefore, work hand-in-hand between GPs and specialists is urged to improve patient care, especially in an era of COVID-19 where hospital overload is detrimental.

## Supplementary Material

Supplemental Material
