# Supplementary material for: Management and characteristics of patients suffering from Clostridiodes difficile infection in primary care
Source: Eur J Gen Pract. 2021 Nov 10;27(1):320–5. doi: 10.1080/13814788.2021.1998447 (PMC8583832; doi:10.1080/13814788.2021.1998447)
Supplement: Supplemental Material [file IGEN_A_1998447_SM7504.docx]

If any of your patients have presented an episode of *Clostridium difficile* infection (ICD) in the last 24 months, thank you for answering the questionnaire concerning this patient. These responses could thus improve the management of community-associated ICD.

* Mandatory answer

1. First, about the patient, his gender, age:*

- Male
- Female
- Age > 65
- age 30-65 years
- age < 30 years

1. Which symptom(s) prompted you to look for an ICD in this patient? *

- Febrile diarrhoea> 38° or biological inflammatory syndrome
- Diarrhea under current antibiotic treatment or within 10 days
- Presence of mucus and/ or blood in the stool
- Abdominal pain/ dyspepsia
- Prolonged diarrhoea > 3 days despite symptomatic treatment
- Other

If other, please specify: _____

1. Is it? *

- First episode of *Clostridium difficile* associated diarrhoea
- A control stool culture after a treatment
- Recurrence

If recurrences, which episode, please specify: _______

1. The diagnosis of ICD was carried out via:

- Search for toxin A/ B/ binary
- Performing a PCR
- Antigen test (GDH)
- Toxinogenic culture
- Lack of bacteriological diagnosis, clinical diagnosis only
- Stool sample culture test performed in the hospital
- Don't know

1. Among these risk factors for ICD, which are attributable to your patient?

- History of inflammatory intestinal disease
- Immunosuppression
- Hospitalization in the last 3 months
- None
- Taking medication, including:
- Proton pump inhibitors
- NSAIDs
- Laxatives or antidiarrheal agents
- Antibiotics

If taking antibiotics, specify the INN if possible: ___

1. Have you had the need to use a specialist opinion?

- Yes
- No

1. What treatment (s) against *Clostridium difficile* has benefited your patient? *

- Therapeutic abstention
- Stopping concomitant antibiotic therapy
- Metronidazole
- Oral vancomycin
- Oral teicoplanin
- Fidaxomicin
- Other

Other, please specify ____

1. On what criteria did you decide on this treatment, or no treatment?

- Presence of severity factors (biological inflammatory syndrome, comorbidities, ...)
- Good clinical tolerance of diarrhoea
- Stopping/ decrease the frequency of diarrhoea
- For practical reasons/ cost of treatment

9. Did the diarrhea stop after?

- 24H
- 48H
- > 48H
- Don't know

10. What is the clinical issue of your patient?*

- Recovery without complications
- Recurrence (reappearance of diarrhea) in the month following the 1st episode
- New episode beyond one month
- Hospitalisation
